# The Effect of Ophiopogonin C in Ameliorating Radiation-Induced Pulmonary Fibrosis in C57BL/6 Mice: An Update Study

**DOI:** 10.3389/fonc.2022.811183

**Published:** 2022-03-30

**Authors:** Xiaobin Fu, Tingting Li, Qiwei Yao

**Affiliations:** ^1^ Department of Radiation Oncology, The Second Affiliated Hospital of Fujian Medical University, Quanzhou, China; ^2^ Department of Radiation Oncology, Fujian Medical University Cancer Hospital & Fujian Cancer Hospital, Fuzhou, China

**Keywords:** radiation-induced pulmonary fibrosis, C57BL/6 mice, OP-C, Chinese medicine, dexamethasone

## Abstract

**Background:**

The aim of this study was to assess and update the protective effects and underlying mechanisms of Ophiopogonin C (OP-C), a biologically active component separated and purified from *Ophiopogon japonicus*, in ameliorating radiation-induced pulmonary fibrosis in C57BL/6 mice administered thoracic radiation.

**Methods and Materials:**

We randomly divided 75 mice into five groups and administered a dose of 12-Gy whole thoracic radiation to establish a pulmonary fibrosis animal model. Mice were treated with OP-C or dexamethasone combined with or without cephalexin by daily gavage for 4 weeks. All mice were sacrificed after the completion of thoracic irradiation at 28 weeks. Serum levels of interleukin-6 and transforming growth factor-β1 (TGF-β1) were evaluated. Moreover, superoxide dismutase (SOD) levels in lung tissue were measured. The severity of fibrosis was evaluated using the hydroxyproline content of the lung tissue. The pathological changes in the five groups were detected by hematoxylin and eosin and Masson trichrome staining. Smooth muscle actin expression was detected using immunohistochemical staining. Matrix metalloproteinases-2 (MMP-2) and tissue inhibitors of metalloproteases-2 (TIMP-2) were examined by immunohistochemical staining of the lung sections, and semiquantitative analysis was used to calculate the expression of MMP-2 and TIMP-2.

**Results:**

Irradiated mice treated with OP-C or DXE combined with or without cephalexin significantly reduced mortality in mice and fibrosis levels by 1) reducing the deposition of collagen and accumulation of inflammatory cells and fibroblasts, 2) downgrading levels of the promote-fibrosis cytokine TGF-β1, and 3) increasing SOD activity in the lung tissue compared with that of irradiated mice without treatment. However, there were no statistical differences in fibrosis levels among the irradiated mice treated with OP-C or DXE combined with or without cephalexin.

**Conclusion:**

OP-C significantly ameliorates radiation-induced pulmonary fibrosis and may be a promising therapeutic strategy for this disorder.

## Background

The incidence of thoracic malignant tumors, such as lung, breast, and esophageal cancers, and thymoma, has been increasing annually in the past decades, and thoracic radiotherapy is the main treatment method for these cancers (1). Radiation-induced lung injury in the normal lung tissue adjacent to the tumor, which includes acute pulmonary inflammation and chronic pulmonary fibrosis, significantly affects the patients’ quality of life, limits the deliverable radiation dose, interrupts radiation treatment, and can lead to death (2, 3). At present, the underlying mechanism of radiation-induced pulmonary injury is not comprehensively understood. It is generally believed that radiation disrupts alveolar epithelial cells and epithelial integrity, leading to edema and damage, which recruit inflammatory cells to release various cytokines, such as interleukin-6 (IL-6), transforming growth factor-β1 (TGF-β1), and tumor necrosis factor-α (TNF-α). A cascade of molecular events alters the microenvironment, and oxidative stress occurs immediately. Furthermore, the proliferation of fibroblasts and the deposition of collagen fibers lead to chronic pulmonary fibrosis, causing progressive fibrosis and insufficient respiratory function (4–6).

The molecular mechanism of radiation-induced pulmonary injury is complicated and unclear; therefore, there are no specific treatment methods. Conventional therapies for radiation-induced pulmonary injury include symptomatic treatment, including steroids, non-steroid anti-inflammatory medicine, antibiotics, and immunosuppressive agents. However, steroid therapy has more side effects, including moon face, high blood pressure, diabetes mellitus, osteoporosis, weakened immune system, and digestive tract ulcers. Moreover, the effects of steroid therapy are unsatisfactory.

Ophiopogonin C (OP-C) is a biologically active component separated and purified from *Ophiopogon japonicus*, which has been widely used as a traditional Chinese medicine for the treatment of inflammatory diseases for a long time. It has also been reported that OP-C plays a role in blocking tumor growth and reducing the cytotoxic effect of chemoradiotherapy (7, 8). In 2019, our group published the role of OC-P in inhibiting acute pulmonary inflammation in mice. The results of this study support the use of OP-C to inhibit radiation-induced acute pulmonary inflammation. However, the effect of OC-P in ameliorating chronic radiation-induced pulmonary fibrosis remains unclear (9). The aim of this study was to assess and update the protective effects and underlying mechanisms of OP-C in ameliorating chronic radiation-induced pulmonary fibrosis in a C57BL/6 mouse model of pulmonary fibrosis.

## Methods and Materials

### Animals

Male inbred C57BL/6 mice, aged 8 weeks and weighing 18–22 g, were purchased from the SLAC Experimental Animal Center (Shanghai, China). They were housed in cages in a specific pathogen-free (SPF) graded animal care facility. Ethical approval for this study was obtained from the Fuzhou General Hospital, Fujian, China. All experimental procedures were performed under general anesthesia, which minimized the pain of the animals. The mice were allowed free access to standard mouse food and water. All mice were housed in cages in a well-controlled SPF-graded animal care facility with a 12/12-h light/dark cycle to acclimate for 7 days prior to the experiment and observed once daily for 28 weeks after whole thoracic irradiation.

### Irradiation and Treatment

Mice were irradiated with 12 Gy using a medical linear accelerator (Trilogy, Varian, CA, USA) at a dose rate of 200 cGy/min. Ten mice were treated at a time using specially designed plexiglass containers to confine the radiation beam. The radiation beam was restricted to the whole thorax. For thoracic irradiation, all mice were anesthetized using 1% sodium pentobarbital (0.8 ml/100 g) intraperitoneally. Mice were observed once daily for up to 28 weeks after radiation, and the body weight and normal death of the mice were recorded. The mice that received thoracic radiation were treated with OP-C (3 mg/kg; Quanzhou Dongnan Traditional Chinese Medicine Co. Ltd., Fujian, China) (n = 15), dexamethasone ([DEX], 1,233 μg/kg; Zhejiang Asia-Pacific Pharmaceuticals Co. Ltd., Zhejiang, China) (n = 15), or DEX + cephalexin (246,600 μg/kg; Zhejiang Asia-Pacific Pharmaceuticals Co. Ltd.) (n = 15) by gavage for 4 weeks after irradiation. The radiation mice (radiation only; n = 15) received thoracic radiation and isotonic sodium chloride solution without drug treatment, but normal saline by gavage. The control group mice (n = 15) were treated with normal saline by gavage at the same volume.

### Study Group

All mice (n = 75) were randomly divided into five experimental groups prior to the experiment as follows: group 1 (blank control group; received no treatment and normal saline gavage); group 2 (radiation-only group; received radiation and normal saline gavage); group 3 (OP-C group; received radiation and OP-C gavage); group 4 (DEX group; received radiation and DEX gavage); and group 5 (DEX + cephalexin group; received radiation and DEX + cephalexin gavage).

### Sample Collection and Histopathology Process

After body weight measurement, all mice were sacrificed at 28 weeks after completion of whole thoracic irradiation. Blood samples were collected from cardiac puncture and kept for 1 h at 25 degrees Celsius (°C) for clotting. Serum samples were collected by centrifugation at 1,000 rpm for 15 min at 2–8°C and then stored at -70°C for further assessment of IL-6 and TGF-β1. The wet weights of both lungs were weighed and recorded. The left lungs were snap frozen with liquid nitrogen and kept at -70°C for further analysis of hydroxyproline (Hyp) and malondialdehyde (MDA) contents and superoxide dismutase (SOD) activity. The right lungs were fixed in 10% formalin solution for 24 h and embedded in paraffin for subsequent histological examination and matrix metalloproteinase-2 (MMP-2) and tissue inhibitors of metalloprotease-2 (TIMP-2) examination by immunohistochemical analyses.

### Histopathological Evaluation

According to the simple method of estimating the severity of pulmonary fibrosis by the Ashcroft et al. (10) scoring system, lung sections stained by hematoxylin and eosin staining and Masson staining and combined with SMA immunohistochemical staining were evaluated. Each group received 3 slides. Each slide received four fields, and the mean value of the four fields was considered representative of the slide. Experienced pathologists in our hospital reviewed and examined the fields. The mean score of all fields was performed as the Ashcroft fibrosis score.

To assess the degree of inflammation in the five groups, the sections stained by hematoxylin and eosin were also reviewed. The pathologists evaluated the accumulation of lymphocytes and neutrophils, interstitial edema, and alveolar wall thickening and classified it into 4 grades on a scale of 0 (absent) to 3 (extensive damage) (score 0: absent, score 1: minimal damage, score 2: severe damage, score 3: extensive damage), as described previously (11).

### Serum Cytokine Level Measurement

Serum levels of IL-6 and TGF-β1 were assessed by ELISA using IL-6 and TGF-β1 kits according to the manufacturer’s instructions (Boster Biological Technology Co. Ltd., Wuhan, China). The optical density values were measured at 450 nm using an ELISA reader and calculated at the linear portion of the curve.

### Collagen Content of Lung Measurement

The lung collagen content was measured using the Hyp assay according to the manufacturer’s instructions (Nanjing Jiancheng Bioengineering Institute, Nanjing, China). Briefly, 30–100 mg of lung tissue was hydrolyzed in lysis buffer solution (approximately 1 ml) at 95°C for 20 min. Finally, the absorbance of the colored lung samples was evaluated at 550 nm.

### SOD Activity Measurement in Lungs

SOD activity was measured using the xanthine oxidase method in the SOD activity assay, according to the manufacturer’s instructions (Nanjing Jiancheng Bioengineering Institute).

### MDA Content Measurement in Lungs

MDA is the end product of reactive oxygen species (ROS)-induced peroxidation of cell membrane lipids, which are reliable markers of oxidative stress. MDA content was measured using the thiobarbituric acid method using an MDA content kit according to the manufacturer’s instructions (Nanjing Jiancheng Bioengineering Institute).

### Masson Trichrome Staining

Masson staining was performed to identify the expression of collagen fibers in lung tissues. Collagen fibers were stained using the Masson staining kit according to the manufacturer’s instructions (Medical Discovery Leader, Beijing, China).

### Immunohistochemical Analyses

Immunohistochemical analyses were performed to identify the expression of MMP-2 and TIMP-2 in the lungs. Briefly, lung sections (5 μm) were incubated with anti-MMP-2 or anti-TIMP-2 primary antibody (1:100 dilution; Santa Cruz Biotechnology, TX, USA) at 4°C overnight. After washing with PBS three times, the sections were incubated with secondary antibodies at 25°C for 50 min and visualized using diaminobenzidine (DAKO, Glostrup, Denmark). SMA expression was also identified by immunostaining. Integrated optical density was calculated using Image-Pro Plus (Media Cybernetics, MD, USA), and the positive area was compared with the total area for semiquantitative analysis.

### Statistical Analysis

Data were analyzed using SPSS version 19.0 (SPSS Inc., IL, USA). Data are expressed as mean ± standard error of mean (SEM). The differences among groups were calculated using the Kruskal–Wallis H and one-way analysis of variance (ANOVA). Statistical significance was set at p < 0.05.

## Results

### OP-C Reduces Mortality in Mice

All C57BL/6 mice irradiated with a single dose of 12 Gy survived after completion of irradiation. The mice tolerated the prescribed dose well. Approximately 50% of mice died at 22–28 weeks after thoracic irradiation. The numbers of surviving mice 28 weeks after irradiation in the blank control, radiation-only, OP-C, DEX, and DEX + cephalexin groups were 13, 5, 7, 8, and 7, respectively. The mortality rates of the five groups were 86.7%, 33.3%, 46.7%, 53.3%, and 46.7%, respectively. Irradiated mice treated with OP-C had a lower mortality rate than irradiated mice without treatment. However, the mortality rate of the irradiated mice treated with OP-C was similar to that of the groups treated with DEX and DEX + cephalexin.

### OP-C Reduces Collagen Deposition in Lung Tissues

**Figure 1:**
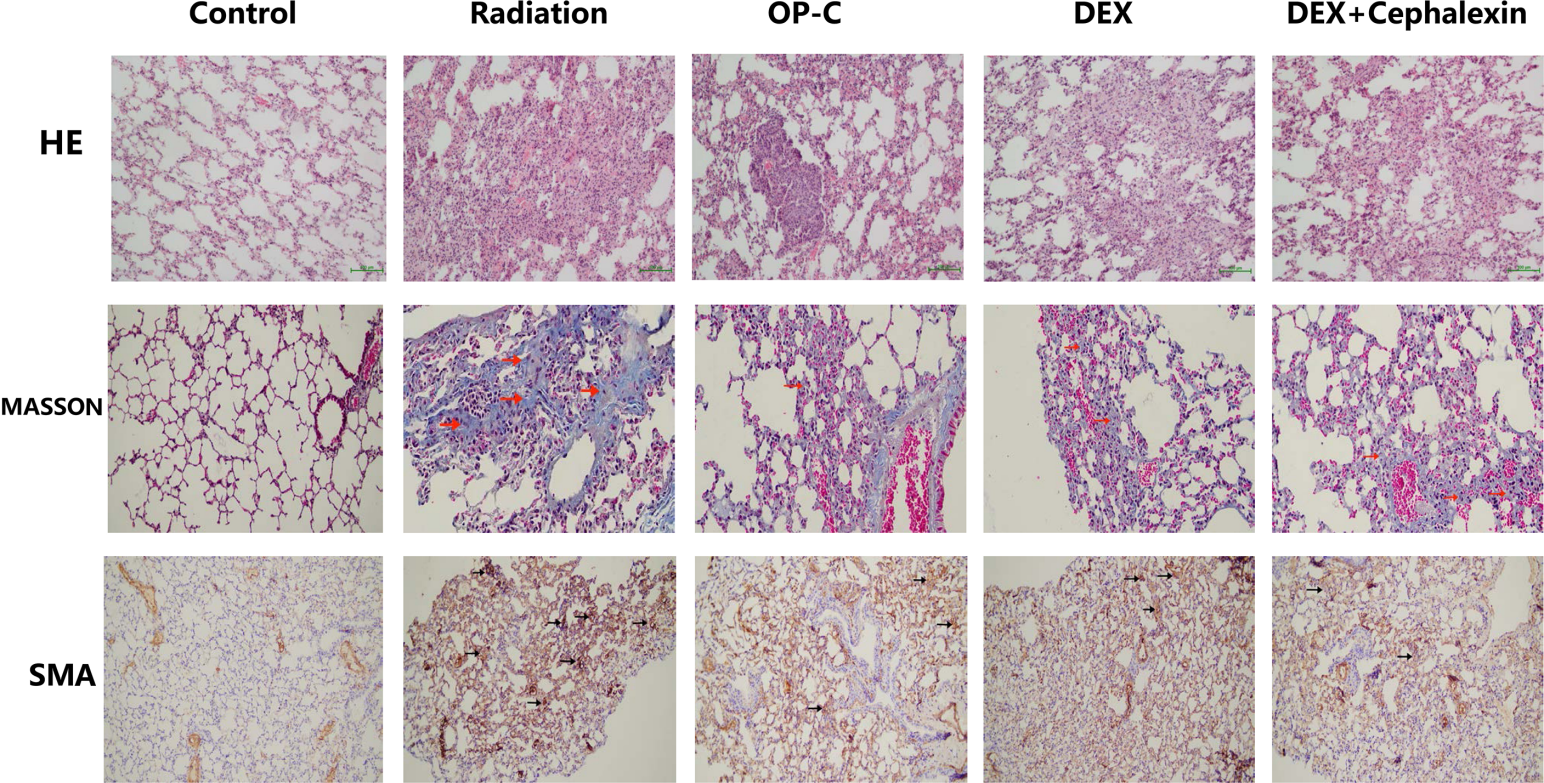
Effects of Ophiopogonin C (OP-C) on the histological changes, including pulmonary inflammation and fibrosis, in the lung tissue at 28 weeks after whole thoracic irradiation. Photomicrographs show staining of mouse lung tissue sections with hematoxylin and eosin staining, Masson staining, and SMA detected by immunohistochemical staining in mice from the blank control, radiation-only, OP-C, dexamethasone (DEX), and DEX + cephalexin groups.

To examine the effect of OP-C on histological changes and collagen deposition in lung tissue, we performed hematoxylin and eosin staining and Masson staining, and SMA was detected by immunohistochemical staining. Irradiated mice without treatment showed chronic moderate pulmonary inflammation changes, including accumulation of lymphocytes and neutrophils, markedly thickened alveolar walls, regional fibrotic foci, accumulation of fibroblasts, and deposition of collagen. Irradiated mice treated with OP-C, DEX, and DEX + cephalexin slightly reduced tissue damage, collagen deposition, and accumulation of inflammatory cells and fibroblasts (**Figure 1**).

**Figure 2:**
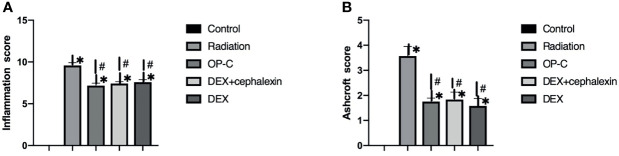
Effects of OP-C on inflammation score **(A)** and Ashcroft score **(B)** in the lungs were measured in mice from the blank control, radiation only, OP-C, DEX, and DEX + cephalexin groups. The presented data are expressed as the mean ± SEM. *p < 0.05, compared with the control group. ^#^p < 0.05, compared with the radiation-only group.

To examine the effect of OP-C on degree of pulmonary inflammation in lung tissue, we used the inflammation scoring system, and we found that the inflammation score was significantly lower in irradiated mice treated with OP-C (7.17 ± 0.52) compared with that of irradiated mice without treatment (9.58 ± 0.58, p < 0.001). However, the Ashcroft score in irradiated mice treated with OP-C was not significantly different from that in mice treated with DEX (7.42 ± 0.38, p = 0.01) or DEX + cephalexin (p = 7.58 ± 0.52, p = 0.01; **Figure 2A**). To examine the effect of OP-C on severity of pulmonary fibrosis in lung tissue, we assessed the Ashcroft scoring system, and we found that the Ashcroft score was significantly lower in irradiated mice treated with OP-C (1.75 ± 0.25) compared with that of irradiated mice without treatment (3.75 ± 0.66, p = 0.001). However, the Ashcroft score in irradiated mice treated with OP-C was not significantly different from that in mice treated with DEX (1.58 ± 0.52, p = 0.01) or DEX + cephalexin (p = 1.83 ± 0.52, p = 0.02; **Figure 2B**).

### OP-C Modulates Serum Cytokine Levels

**Figure 3:**
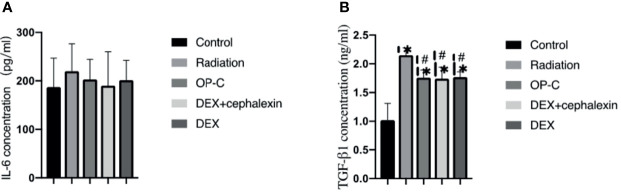
Effects of OP-C on cytokine expression in serum. Expression of IL-6 **(A)** and TGF-β1 **(B)** in the serum were measured in mice from the blank control, radiation only, OP-C, DEX, and DEX + cephalexin groups. The presented data are expressed as the mean ± SEM. *p < 0.05, compared with the control group. ^#^p < 0.05, compared with the radiation-only group.

To examine the effect of OP-C on the expression of serum cytokines, we assessed serum IL-6 and TGF-β1 levels using ELISA. No significant differences in IL-6 expression were seen in the control mice (187.15 ± 59.13 pg/ml), irradiated mice without treatment (220.05 ± 56.62 pg/ml), irradiated mice treated with OP-C (203.09 ± 41.4 pg/ml), irradiated mice treated with DEX (190.34 ± 69.66 pg/ml), and irradiated mice treated with DEX + cephalexin (201.71 ± 40.71 pg/ml, p = 0.765) (**Figure 3A**). We found that serum TGF-β1 expression was significantly lower in irradiated mice treated with OP-C (1.76 ± 0.13 ng/ml) compared with that of irradiated mice without treatment (2.15 ± 0.13 ng/ml, p = 0.046). Moreover, TGF-β1 expression was also significantly lower in irradiated mice treated with DEX (1.77 ± 0.09 ng/ml, p = 0.05) and DEX + cephalexin (1.74 ± 0.2 ng/ml, p = 0.04) compared with that of irradiated mice without treatment. However, the level of serum TGF-β1 in irradiated mice treated with OP-C was not significantly different from that in mice treated with DEX (p = 0.972) or DEX + cephalexin (p = 0.992; **Figure 3B**).

### OP-C Reduces Hyp Content in the Lungs

**Figure 4:**
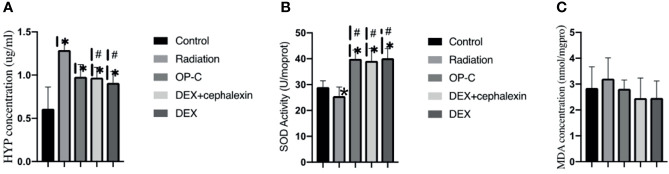
Effects of OP-C on hydroxyproline (Hyp) contents, superoxide dismutase (SOD) activity, and malondialdehyde (MDA) contents in the lungs. Expression of Hyp **(A)**, SOD activity **(B)**, and MDA contents **(C)** in the lungs were measured in mice from the blank control, radiation-only, OP-C, DEX, and DEX + cephalexin groups. The presented data are expressed as the mean ± SEM. *p < 0.05, compared with the control group. ^#^p < 0.05, compared with the radiation-only group.

To examine the effect of OP-C on collagen deposition, we measured the Hyp content of the left lungs. We found that the expression of Hyp content was lower in irradiated mice treated with OP-C (0.98 ± 0.14 μg/ml) than in irradiated mice without treatment (1.29 ± 0.1 μg/ml, p = 0.082); however, statistical analysis showed no significant difference. The expression of Hyp content was significantly lower in irradiated mice treated with DEX (0.91 ± 0.13 μg/ml, p = 0.008) and DEX + cephalexin (0.97 ± 0.12 μg/ml, p = 0.04) compared with that of irradiated mice without treatment. However, the level of Hyp content in irradiated mice treated with OP-C was not significantly different from that in mice treated with DEX (p = 0.425) or DEX + cephalexin (p = 0.937; **Figure 4A**).

### OP-C Modulates SOD Activity and MDA Content in the Lungs

To examine the effect of OP-C on redox balance, we assessed the SOD activity and MDA content in the lungs. We found that SOD activity was significantly higher in irradiated mice treated with OP-C (39.98 ± 3.38 U/ml) compared with that of irradiated mice without treatment (25.57 ± 3.48 U/ml, p = 0.001). Moreover, SOD activity was significantly higher in irradiated mice treated with DEX (40.17 ± 3.78U/ml, p = 0.001) and DEX + cephalexin (39.18 ± 4.79 U/ml, p = 0.001) compared with that of irradiated mice without treatment. However, SOD activity in irradiated mice treated with OP-C was not significantly different from that in mice treated with DEX (p = 0.925) or DEX + cephalexin (p = 0.906; **Figure 4B**). No significant differences in the expression of MDA content were observed in the control mice (2.85 ± 0.82 nmol/ml), irradiated mice without treatment (3.21 ± 0.81 nmol/ml), irradiated mice treated with OP-C (2.82 ± 0.34 nmol/ml), irradiated mice treated with DEX (2.47 ± 0.65 nmol/ml), and irradiated mice treated with DEX + cephalexin (2.46 ± 0.78 nmol/ml, p = 0.377; **Figure 4C**).

### OP-C Modulates MMP-2 and TIMP-2 Content in the Lungs

**Figure 5:**
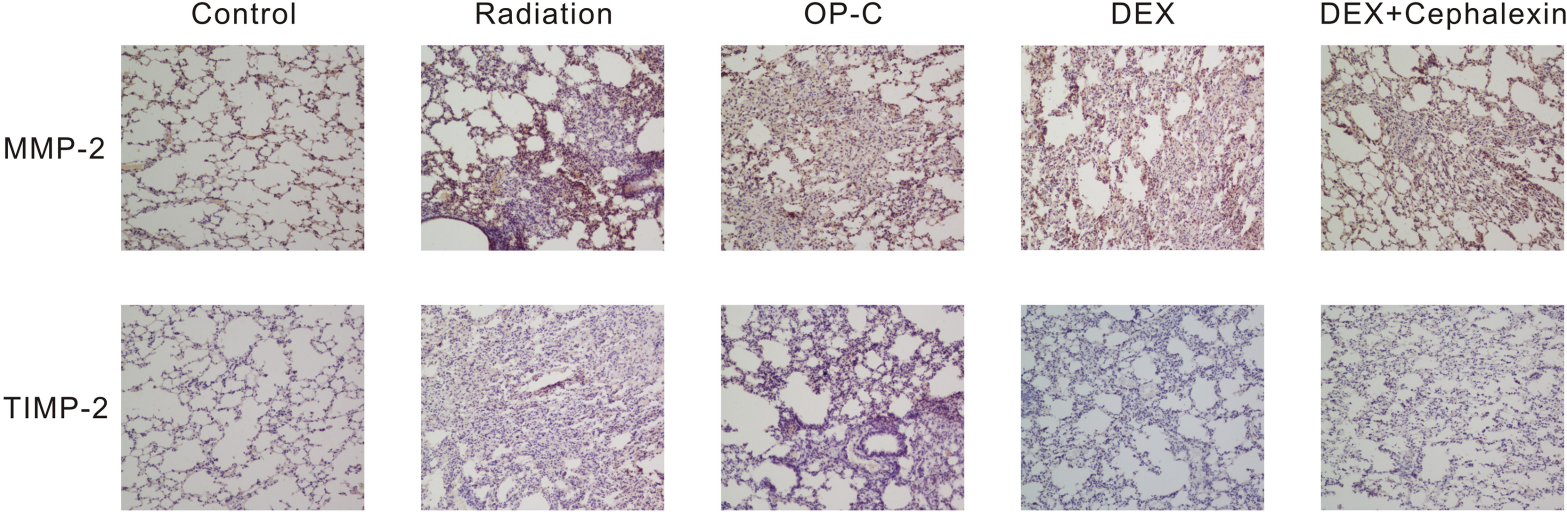
Effects of Ophiopogonin C (OP-C) on the expression of MMP-2 and TIMP-2 changes in the lung tissue at 28 weeks after whole thoracic irradiation. Photomicrographs show staining of mouse lung tissue sections with MMP-2 and TIMP-2 detected by immunohistochemical staining in mice from the blank control, radiation-only, OP-C, dexamethasone (DEX), and DEX + cephalexin groups.

**Figure 6:**
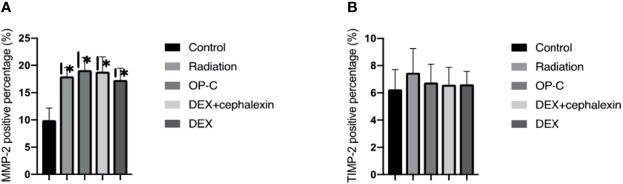
Effects of OP-C on the expression of matrix metalloproteinases-2 (MMP-2) **(A)** and tissue inhibitors of metalloproteases-2 (TIMP-2) in the lungs **(B)**. Expressions of MMP-2 and TIMP-2 in the lungs were measured in mice from the blank control, radiation-only, OP-C, DEX, and DEX + cephalexin groups. The presented data are expressed as the mean ± SEM. *p < 0.05, compared with the control group. ^#^p < 0.05, compared with the radiation-only group.

To examine the effect of MMP-2 and TIMP-2 content in the lungs, we performed immunohistochemical analyses and calculated the positive area by semiquantitative analysis (**Figure 5**). We found that the MMP-2 content was significantly lower in control mice (9.95 ± 2.22%) than in irradiated mice treated with OP-C (19.11 ± 2.37%, p < 0.001). However, MMP-2 expression in the irradiated mice without treatment (17.98 ± 1.62%), irradiated mice treated with OP-C (19.11 ± 2.37%), irradiated mice treated with DEX (17.33 ± 2.13%), and irradiated mice treated with DEX + cephalexin (18.84 ± 2.73%) was not significantly different (p = 0.792; **Figure 6A**). Moreover, no significant differences in the expression of TIMP-2 content were seen in the control mice (6.26 ± 1.45%), irradiated mice without treatment (7.48 ± 1.78%), irradiated mice treated with OP-C (6.76 ± 1.35%), irradiated mice treated with DEX (6.63 ± 0.94%), and irradiated mice treated with DEX + cephalexin (6.6 ± 1.27%, p = 0.794; **Figure 6B**).

## Discussion

In our study, we demonstrated that OP-C, a biologically active component found in *Ophiopogon japonicus*, ameliorated radiation-induced pulmonary fibrosis in C57BL/6 mice treated with a single dose of 12-Gy whole thoracic radiation. In irradiated mice, OP-C treatment significantly reduced the mortality rate, slightly alleviated lung histological tissue damage, including decreased accumulation of inflammatory cells and proliferation of fibroblasts, and inhibited collagen deposition. OP-C also regulated the levels of promote-fibrosis serum TGF-β1 and Hyp content. Moreover, we demonstrated that OP-C treatment significantly increased the activity of antioxidant enzymes in SOD. However, the levels of pulmonary fibrosis were not significantly different among the irradiated mice treated with OP-C, DEX, and DEX + cephalexin. We demonstrated that OP-C had similar protective effects to steroid therapy against radiation-induced pulmonary fibrosis. Moreover, compared with steroid therapy, no obvious adverse effects were found with OP-C treatment. Thus, OP-C may be a promising therapeutic strategy for radiation-induced pulmonary fibrosis.

TGF-β1 is known as a promote-fibrosis cytokine, which has a direct effect on fibroblasts to promote fibroblast division and proliferation and the synthesis and deposition of extracellular matrix (ECM) proteins, and enhances the expression of relevant receptors of ECM, thereby promoting the development of pulmonary fibrosis (12–14). Previous studies have demonstrated that serum cytokine TGF-β1 could be treated as a predictive factor for pulmonary fibrosis. Rubin et al. (15) studied the relationship between serum cytokine TGF-β1 and collagen deposition in C57BL/6 mice treated with a single dose of 12.5-Gy thoracic radiation and found that there was no significant change in TGF-β1 expression at 8 weeks post-irradiation. However, TGF-β1 expression was significantly increased at 8–24 weeks post-irradiation. TGF-β1 also had a positive correlation with collagen deposition. Robb et al. (16) studied the effects of amino acid taurine in attenuating lung fibrosis in C57BL/6 mice treated with a single dose of 14-Gy thoracic radiation and found that amino acid taurine significantly decreased the serum TGF-β1 levels and Hyp content at 14 weeks post-irradiation. In our study, C57BL/6 mice received 12-Gy thoracic radiation and were treated with OP-C by gavage. The expression of serum TGF-β1 was significantly lower in irradiated mice treated with OP-C than in irradiated mice without treatment.

Hyp, which accounts for 13.4% of collagen proteins, is one of the main components of collagen proteins and is not found in other proteins. Moreover, collagen proteins are the main components of pulmonary and hepatic fibrosis. Therefore, Hyp is a sensitive predictive biomarker to determine the levels of pulmonary and hepatic fibrosis (17). Zhou et al. (18) studied the effects of lettuce glycoside B in ameliorating pulmonary fibrosis in Sprague-Dawley rats that received a single dose of 22-Gy whole thoracic radiation and found that treatment with lettuce glycoside B significantly decreased the content of Hyp in irradiated rats compared with that of irradiated rats without treatment, which is similar to reports by Hua You et al. Hua You et al. (19) studied male Sprague-Dawley rats that received a single dose of 22-Gy whole thoracic radiation and found that the irradiated rats administered the green extract epigallocatechin-3-gallate (EGCG) had a significantly decreased Hyp content 120 days post-irradiation compared with that of the irradiated rats without treatment. EGCG treatment significantly inhibited radiation-induced pulmonary fibrosis. In our study, the irradiated mice treated with OP-C had a lower Hyp content compared with that of the irradiated mice without treatment; however, the difference was not statistically significant.

Numerous studies have revealed that ROS and oxidant stress have a direct and indirect impact on radiation-induced pulmonary fibrosis. ROS immediately activate inflammatory cells, including neutrophils, monocytes, and lymphocytes, and lead to a positive feedback loop that increases the expression of oxidative enzymes and the synthesis of ROS. Thus, the balance of oxidant/antioxidant was damaged and the normal tissue damage and deposition of collagen persisted. Certain studies have demonstrated that treatment methods, including defending against and alleviating oxidative stress, could be a promising treatment strategy for ameliorating radiation-induced pulmonary fibrosis. SOD activity and MDA content in serum or lung tissue are crucial and sensitive biomarkers of oxidative stress and the response to oxidative stress. SOD plays a crucial role in maintaining the oxidant/antioxidant balance, which catalyzes and neutralizes the free-radical form of oxygen to generate hydrogen peroxide. MDA content reflects the levels of organic lipid peroxidation, which reveals the degree of cell membrane damage (20–22). Kang et al. (23) studied extracellular SOD-overexpressing B6C3 transgenic mice that received thoracic radiation and demonstrated that the overexpression of extracellular SOD transgenic mice significantly decreased oxidative stress and radiation-induced pulmonary injury compared with irradiated wild-type mice. SOD may be a promising treatment agent for radiation-induced pulmonary injuries. In another study by Pan et al. (24), irradiated Kunming mice received SOD-TAT, a fusion protein of the HIV-1Tat protein transduction domain and hCuZn-SOD, which significantly enhanced the pulmonary antioxidant ability and ameliorated radiation-induced pulmonary fibrosis compared with irradiated mice treated with amifostine and irradiated mice without treatment. Liu et al. (25) studied the protective effects of quercetin liposomes against radiation-induced pulmonary injury and found that irradiated C57BL/6 mice treated with quercetin liposomes had significantly increased SOD activity and decreased MDA content to protect against radiation-induced acute pneumonia and chronic pulmonary fibrosis by reducing oxidative damage compared with irradiated mice without treatment. In our study, irradiated mice treated with OP-C showed significantly increased SOD activity in the lung tissue to relieve pulmonary fibrosis by decreasing the oxidant stress compared with irradiated mice without treatment. However, no significant differences in the expression of MDA content were observed in our study.

The MMP family, a family of zinc-dependent endopeptidases, are highly homologous and possess over 20 family members. CD147, an MMP inducer, is synthesized to increase MMP expression during lung disease development. Certain studies have demonstrated that MMPs play a crucial role in degrading the ECM and remodeling structural proteins, including collagens and elastin. MMPs then restructure normal lung tissue to initiate the pathogenesis of lung diseases. The TIMP family, MMPs’ endogenous inhibitors, comprise four related members, namely, TIMP-1, -2,-3, and -4, which lead to dual control to inhibit the active form and activation process of MMPs. The balance, activation, and normal expression of MMPs and TIMPs in normal lung tissue are tightly regulated to prevent harmful effects on normal lung tissue. The upregulation of MMPs and TIMPS is observed in initial lung disease development, lung tissue remodeling, and pulmonary fibrosis (26, 27). MMP-2, a critical member of MMPs, has a high affinity for collagen IV of the basement membrane to degrade type IV and remodel the pulmonary structure. Yang et al. (28) studied the effect of thoracic radiation on the MMP/TIMP system in normal lung tissue and found that thoracic radiation significantly increased the expression of MMP-2 and MMP-9 to degrade collagen IV, thereby damaging the integrity of normal lung tissue. However, the expression of TIMP-1, -2, and -3 in the lungs was not influenced by thoracic radiation. In another study by Rave-Frank et al. (29), rats received a high single-dose irradiation of 25 Gy, and it was found that the expression of MMP-2, -9, and -14, and TIMP-1, -2, and -3 significantly increased shortly after irradiation, however not at 3 months post-irradiation. Similar to this study, the expression of MMP-2 and TIMP-2 at 28 weeks post-irradiation was not significantly different among the irradiated mice without treatment, treated with OP-C, DEX, and DEX + cephalexin in our study.

This study has several limitations. First, this was a prospective study with a small sample size. A larger-scale study is required to validate these findings. Second, few blood samples were collected from C57BL/6 mice by cardiac puncture. Larger mice or rabbits are recommended for further study. Third, this study demonstrated that OP-C significantly ameliorated pulmonary fibrosis. However, the protective effects of OP-C at different doses are warranted in future studies.

## Conclusion

Overall, the present study demonstrated that OP-C significantly ameliorates radiation-induced pulmonary fibrosis in C57BL/6 mice treated with a single dose of 12-Gy whole thoracic radiation. However, our data demonstrated that OP-C had similar protective effects on pulmonary fibrosis as steroid therapy. No obvious adverse effects were observed in the OP-C treatment. Therefore, OP-C may be a promising therapeutic strategy for this disorder.

## Data Availability Statement

The data sets generated during and/or analyzed during the current study are available from the corresponding author on reasonable request.

## Ethics Statement

The animal study was reviewed and approved by The Fujian Cancer Hospital.

## Author Contributions

All authors listed have made a substantial, direct, and intellectual contribution to the work and approved it for publication.

## Funding

This study was supported in part by grants from the Fujian Provincial Platform for Medical Laboratory Research and Key Laboratory for Tumor Individualized Active Immunity (Project Number: FYKFKT-2017015).

## Conflict of Interest

The authors declare that the research was conducted in the absence of any commercial or financial relationships that could be construed as a potential conflict of interest.

The reviewer YS declared a shared affiliation with two of the authors (XF, TL), with no collaboration to the handling editor at the time of the review.

## Publisher’s Note

All claims expressed in this article are solely those of the authors and do not necessarily represent those of their affiliated organizations, or those of the publisher, the editors and the reviewers. Any product that may be evaluated in this article, or claim that may be made by its manufacturer, is not guaranteed or endorsed by the publisher.
